# Morphological and cytokine profiles as key parameters to distinguish between Gram-negative and Gram-positive bacterial keratitis

**DOI:** 10.1038/s41598-020-77088-w

**Published:** 2020-11-18

**Authors:** Aris Konstantopoulos, Maria del Mar Cendra, Michael Tsatsos, Mariam Elabiary, Myron Christodoulides, Parwez Hossain

**Affiliations:** 1 Southampton Eye Unit, MP104, Southampton General Hospital, University Hospital Southampton NHS Foundation Trust, Tremona Road, Southampton, SO16 6YD UK; 2 Clinical and Experimental Sciences, Southampton General Hospital, University of Southampton, Southampton, UK; 3 Aristotle University of Thessaloniki, Thessaloniki, Greece; 4 Eye Department, Dorset County Hospital NHS Foundation Trust, Dorchester, UK

**Keywords:** Cytokines, Microbiology, Medical research

## Abstract

Bacterial keratitis (BK) is an ocular disorder associated with poor visual prognosis. Quantification of the associated inflammatory response may provide insight into the pathogenesis of BK and guide treatment options. In this exploratory study, we evaluated 45 BK patients and 20 healthy controls by optical coherence tomography and pro-inflammatory tear cytokine analysis. The aim was to quantify the differential morphological and cytokine inflammatory response between Gram-negative and Gram-positive BK and to determine the diagnostic value of corneal thickness (CT) and infiltrate thickness (IT) in distinguishing Gram−ve BK in a clinical cohort. Greater CT and IT, at clinical presentation, were indicative of Gram−ve infection with values detected of ≥ 950 μm and ≥ 450 μm, respectively. Combination of these CT and IT values had a 100% sensitivity and 83.3% specificity as a diagnostic indicator of Gram−ve infection. Similarly, there were higher levels of IL-1β, IL-6 and IL-8 cytokines were quantified in keratitis caused by Gram-negative bacteria. Among the different tear cytokines analysed, a significant reduction after three days of treatment was detected for pro-inflammatory cytokines IL-1β, IL-2, IL-6, IL-8 and TNF-α, prior to starting with the administration of steroid drops. Overall, this study shows the potential value of serial OCT and tear cytokine measurements in the management of BK.

## Introduction

Bacterial keratitis (BK) is the most common corneal infection in the western world^1–3^ resulting in frequent visits to eye casualty worldwide. The condition affects predominantly the working-age population^1–4^ and it is associated with significant morbidity with up to 40% of patients developing poorer visual acuity than at presentation^5^ and 23.3% experiencing loss of two or more Snellen lines of visual acuity (VA) compared to the fellow eye. Bacterial keratitis can be mild with no visual consequences but often is a significant cause of visual impairment with two million cases of monocular blindness annually^4^ (as per the World Health Organisation).

Microbial cultures from corneal scrapes remain the mainstay of microbial identification, but positive cultures are only achieved in 63.8–71.2% of the total number of patients having scrapes and typically requires 48 h^4–6^. In day to day practice, diagnosis of BK is based on slit-lamp examination and photographic documentation without relying on the isolation of the causative pathogen^6–8^. Delay in presentation and treatment and large corneal ulcer size are associated with poor final visual acuity^9^ making prompt diagnosis and treatment essential for the best possible outcome.

Bacterial keratitis caused by Gram-negative (Gram−ve) bacteria has been shown to present with greater inflammatory response and resultant infiltration as assessed on the slit-lamp compared to Gram-positive (Gram+ve) bacteriae^5,6^. Elevated levels of pro-inflammatory cytokines and chemokines, such as IL-1β, IL-6, MIP-2, IL-8 and IFN-γ have been shown in both human and animal studies of BK^10–16^. Presence of pro-inflammatory cytokines in tears has been suggested to play a key role in pathogenesis^17^. Furthermore, elevated levels of IL-1β, IL-6, IL-8 have been detected in bacterial keratitis tears^18,19^. However, the association between these cytokines and bacterial type as well as their levels during the course of the disease remain elusive.

Corneal transparency and integrity rely on the avascular and clear non-inflamed nature of the corneal layers. Prompt treatment of the microbial infection and early control of the resultant inflammation is essential for the preservation of good visual function. The ever-growing problem of microbial resistance and use of contact lenses from a younger age are making bacterial keratitis an increasing problem despite the advent of newer generation anti-microbial agents. The successive corneal inflammation is currently controlled with topical steroid drops irrespective of the types of the bacterial corneal infection, but their effect on the final visual outcome as well as the ideal timing of their initiation and duration of treatment remained undetermined^20^.

In this exploratory study, we aimed to quantify the differential morphological and cytokine inflammatory response between Gram−ve and Gram+ve BK in a clinical setting and examine their association with the diagnostic value of anterior segment optical coherence tomography (AS-OCT) parameters in identifying Gram−ve infection. We have previously demonstrated the quantification capabilities of AS-OCT in providing cross-sectional scans of the cornea and in measuring the stromal infiltration thickness and corneal thickness in the infiltrated area, as well as monitoring in vivo parameters of corneal inflammation in bacterial keratitis^8,9^. By understanding the immune response and pathogenesis of BK, there is potential to develop new therapies to limit the sight-threatening tissue destruction that can occur with an infection.

## Results

### Clinical and optical coherence tomography study

45 BK patients (right/left eyes: 23/22; 21 male and 24 female) were included. Mean [SD] age was 47 [20.6] years. Mean [SD] duration of symptoms at presentation was 3.3 [2.8] days. Microbiology showed 21 g−ve bacteria (18 *Pseudomonas aeruginosa*, 1 *Chryseobacterium*, 1 coliform species, 1 g−ve on microscopy), 13 g+ve bacteria (7 coagulase-negative *Staphylococcus *(> 80%* Staphylococcus epidermidis)*, 3 *Staphylococcus aureus*, 2 diphtheroid species, 1 *Streptococcus pneumoniae*, 1 *Enterococcus* species, 1 *Bacillus* species—2 cases were polymicrobial) and 11 negative cases. Contact lens wear was the most common risk factor (n = 30), followed by recurrent corneal erosion syndrome (n = 4). Age and duration of symptoms were not significantly different in the three groups (Gram−ve, Gram+ve and negative cases; *p* = 0.927 and 0.228, respectively). Mean [SD] follow-up was 28.6 [28.4] days; there was a significant difference between Gram−ve (39.5 [31.7] days) versus Gram+ve (17.9 [12.6] days) (*p* = 0.026) but no difference when each of these group were compared to the negative microbiology cases (22.2 [26.8] days) (*p* = 0.146). Since the Negative cases had, by definition, no positive identified bacterial organism from their corneal sample, this group were subsequently excluded from the study. Twenty healthy volunteers (5 male, 15 female) were recruited as microbiology negative BK controls; the mean [SD] age was 42.7 [10.7] years (age range 27–59). These controls were followed-up over the study period.

Examining visual acuity in BK patients, mean [SD] logMAR visual acuity at presentation was 1.21 [1.22]; this was significantly greatest in Gram−ve BK compared to Gram+ve and microbiology negative BK (1.78 [1.28] versus 0.90 [1.0] vs. 0.53 [0.95], *p* = 0.002) (Table 1). Mean [SD] final logMAR visual acuity was significantly better than presentation logMAR visual acuity (0.33 [0.71] vs. 1.21 [1.22], *p* < 0.001). The individual differences between the groups for visual acuity at presentation and final follow-up are shown in Table 1, Gram−ve presented with worse vision. The mean [SD] presentation epithelial defect (ED) overlying the infiltrate and the infiltrate diameters (ID) were 2.2 [1.5] and 2.1 [1.3] mm, respectively; both were largest in Gram−ve BK (Table 1).

**Table 1:** VA, ED, ID, IT, CT, CTS and CTL comparison between Gram-negative, Gram-positive and microbiology negative keratitis.

Parameter	Gram−ve versus Gram+ve versus Microbiology − ve	*p* value	Post-hoc *p* value Gram−ve versus Gram+ve
Presentation VA-logMAR (mean [SD])	1.78 [1.28] versus 0.90 [1.0] versus 0.53 [0.95]	0.002	0.014
Presentation ED-mm (mean [SD])	2.7 [1.5] versus 2.0 [1.2] versus 0.9 [0.5]	< 0.001	0.138
Presentation ID-mm (mean [SD])	2.7 [1.4] versus 1.5 [0.7] versus 1.1 [0.6]	0.001	0.002
Presentation IT-μm (mean [SD])	560.8 [182.4] versus 304.4 [75.0] versus 301.3 [104.7]	0.001	0.001
Presentation CT-μm (mean [SD])	1147.1 [152.2] versus 840 [104.2] versus 782.7 [69.7]	< 0.001	< 0.001
CTI-μm (mean [SD])	562.3 [159.5] versus 240 [100.5] versus 181.4 [81.9]	< 0.001	< 0.001
Final VA-logMAR (mean [SD])	0.22 [0.45] versus 0.69 [1.11] versus 0.07 [0.16]	0.12	NA
Final CT-μm (mean [SD])	445 [94.9] versus 531.7 [157] versus 607.8 [76.8]	0.003	0.17
CTL-μm (mean [SD])	131.7 [112.5] versus 44.4 [158.6] versus − 6.8 [60.2]	0.011	0.12

*VA* visual acuity, *ED* epithelial defect, *ID* infiltrate diameter, *IT* infiltrate thickness, *CT* corneal thickness, *CTI* corneal tissue inflammation, *CTL* corneal tissue loss.

The mean [SD] AS-OCT corneal thickness (CT) at presentation of BK was 966.7 [223] µm, which was greatest in Gram−ve infection (1147.1 [152.2] vs. 840 [104.2] vs. 782.7 [69.7] µm, *p* < 0.001). In the Gram−ve group, 18 cases had CT ≥ 1000 μm, whereas, in the Gram+ve group, only 1 case had CT ≥ 1000 μm (*p* < 0.001). Within the Gram−ve group, all 21 cases had CT ≥ 950 μm compared to 2 cases in the Gram+ve group (*p* < 0.001). The sensitivity, specificity, positive predictive value (PPV) and negative predictive value (NPV) of CT ≥ 1000 μm as a diagnostic indicator of Gram−ve infection was 85.7%, 95.8%, 94.7% and 84.6% respectively. The sensitivity, specificity, PPV and NPV of CT ≥ 950 μm were 100%, 91.7%, 91.3% and 100% respectively. A criterion of CT ≥ 950 μm or infiltrate thickness (IT) ≥ 400 μm had a sensitivity, specificity, PPV and NPV of 100%, 79.2%, 80.8% and 100%. A criterion of CT ≥ 950 μm or IT ≥ 450 μm had a sensitivity, specificity, PPV and NPV of 100%, 83.3%, 84% and 100% respectively, as a diagnostic indicator of Gram−ve infection. Mean [SD] final CT was significantly less than presentation CT (509 [124.1] vs. 966.7 [223] μm, *p* < 0.001). Final CT was least in Gram−ve BK compared to Gram+ve or microbiology negative BK (445 [94.9] vs. 531.7 [157] vs. 607.8 [76.8] μm, *p* = 0.003; Table 1).

**Figure 1:**
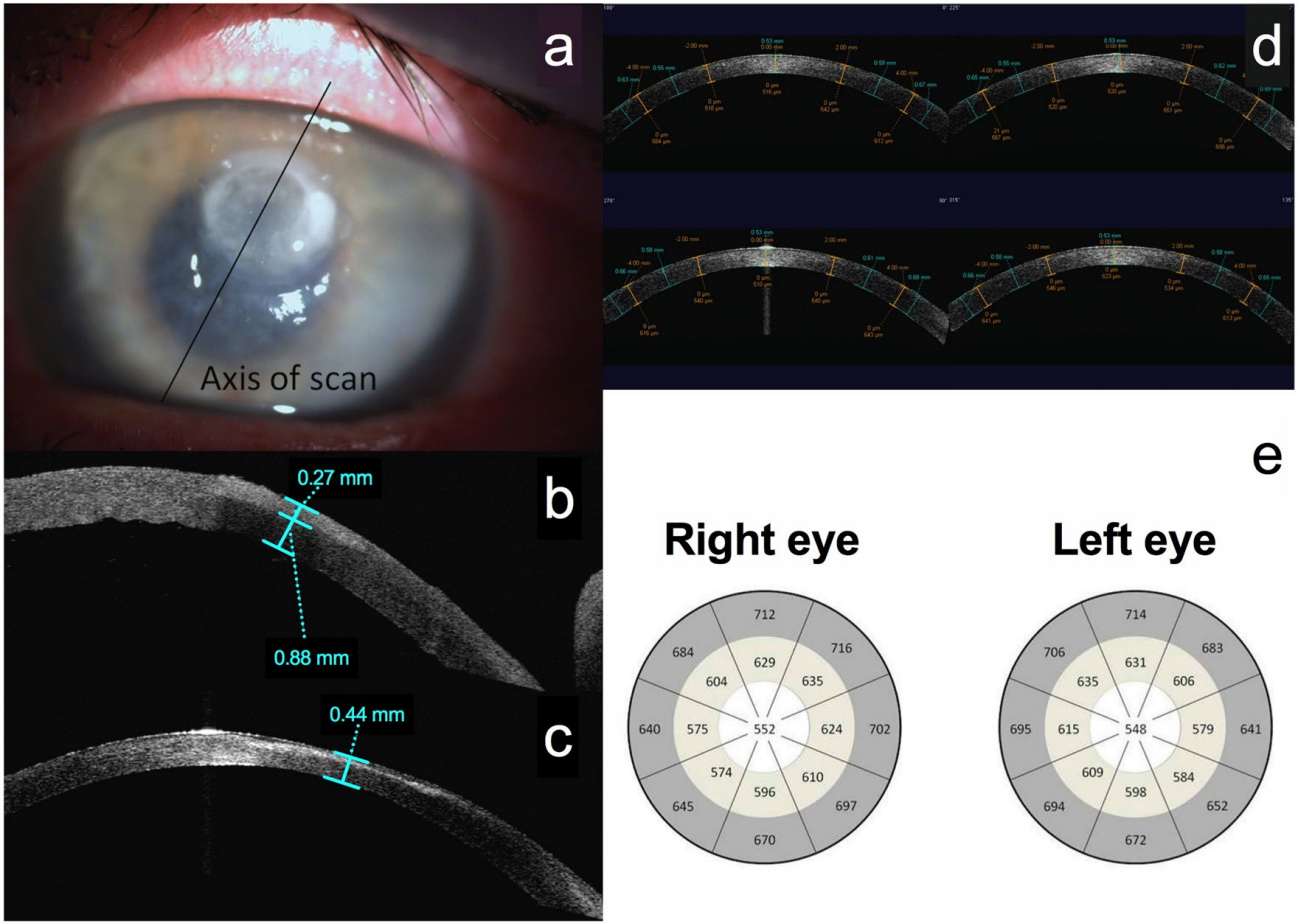
Anterior segment optical coherence imaging protocol for bacterial keratitis (**a**–**c**) and controls (**d**, **e**). (**a**) Picture of bacterial keratitis, illustrating the anterior segment optical coherence tomography (AS-OCT) imaging protocol with the same axis high resolution scan carried out at presentation and after resolution of infection. (**b**) An AS-OCT scan at presentation, illustrating the measurement of corneal thickness (810 μm) and infiltrate thickness (270 μm) at presentation. (**c**) Measurement of final corneal thickness (440 μm) once the infection has resolved. (**d**) Four-quadrant AS-OCT scans of the control healthy cornea. The flap tool was used to identify a central 4 mm area, a mid-peripheral area defined by the 4 mm zone and an outer 8 mm zone, and a peripheral area extending from the 8 mm zone to the limbus. The corneal thickness was measured in the centre of each area on all four scans with calliper tools and the mean corneal thickness of all patients plotted. (**e**) The plotted corneal thickness maps of healthy control subjects.

In the control group, mean [SD] central CT was 552 [36.4] μm right eye and 547.5 [33.1] μm left eye. Figure 1 shows the CT in the centre of each zone and the plotted CT maps for bacterial keratitis and controls. The mean [SD] presentation IT was 414.7 [186.7] μm; this was greatest in Gram−ve BK, compared to Gram+ve or to the microbiology negative BK group (560.8 [182.4] vs. 304.4 [75.0] vs. 301.3 [104.7] µm, *p* = 0.001; Table 1). Measurement of IT was possible (as per scanning protocol^8,9^) in 36 cases; In the Gram−ve group, 16 cases had IT ≥ 400 μm, of whom 13 cases had IT ≥ 450 μm. In the Gram+ve group, only 1 case had IT ≥ 450 μm, and only 1 case had IT ≥ 400 μm. The differences between Gram−ve and Gram+ve groups were significant for both 450 and 400 μm measurement criteria (*p* = 0.003 and *p* < 0.001 respectively). Sensitivity, specificity, PPV and NPV of IT ≥ 400 μm as a diagnostic indicator of Gram−ve infection was 88.9%, 83.3%, 84.2% and 88.2% respectively. Sensitivity, specificity, PPV and NPV of IT ≥ 450 μm were 72.2%, 88.9%, 86.7% and 76.2% respectively.

The mean [SD] corneal tissue swelling (CTS) at presentation was 376.1 [217.6] µm. There was a significant difference between Gram−ve, Gram+ve and microbiology negative BK (562.3 [159.5] vs. 240 [100.5] vs. 181.4 [81.9] µm, *p* < 0.001). This difference was significant between Gram−ve and Gram+ve BK (*p* < 0.001); the ratio of Gram−ve to Gram+ve CTS was 2.3. The difference between Gram−ve and microbiology negative BK was also significant (*p* < 0.001) and the ratio was 3.1. Presentation CTS correlated with logMAR visual acuity (r = 0.610, p < 0.001), IT (r = 0.662, *p* < 0.001), epithelial defect (r = 0.553, *p* < 0.001) and infiltrate diameter (r = 0.693, *p* < 0.001) at presentation, and final CT (r = 0.610, *p* < 0.001) and corneal tissue loss (CTL) (r = 0.547, *p* = 0.002) (Supplementary Table 1).

**Figure 2:**
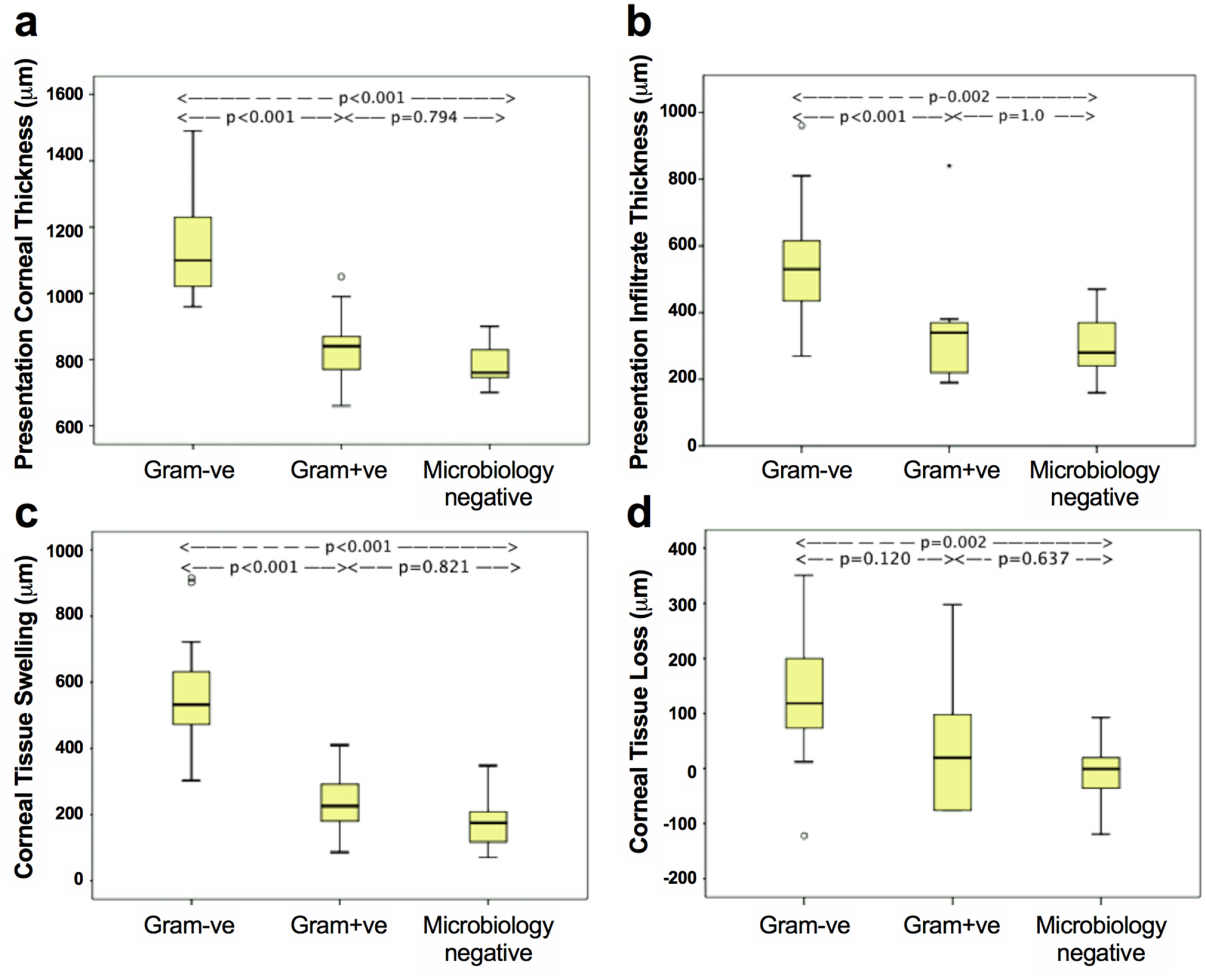
Comparison of AS-OCT quantification parameters between Gram−ve, Gram+ve and microbiology negative bacterial keratitis. (**a**) There was a significant difference in presentation corneal thickness between Gram−ve and Gram+ve groups, and between Gram−ve and microbiology negative groups, but not between Gram+ve and microbiology negative groups. (**b**) There was a significant difference in presentation infiltrate thickness between Gram−ve and Gram+ve BK, and Gram−ve and microbiology negative BK, but not between Gram+ve and microbiology negative BK (*p* = 1.0). (**c**) There was a significant difference in corneal tissue swelling between Gram−ve and Gram+ve groups, and Gram−ve and microbiology negative groups, but not between Gram+ve and microbiology negative groups. (**d**) There was a borderline significant difference in corneal tissue loss between Gram−ve and Gram+ve BK (*p* = 0.12), a significant difference between Gram−ve and microbiology negative BK, and no significant difference between Gram+ve and microbiology negative BK.

The mean [SD] CTL was 75.1 [120.5] µm, which was greatest in Gram−ve infection compared to Gram+ve or to reference microbiology negative group (131.7 [112.5] vs. 44.4 [158.6] vs. − 6.8 [60.2] µm, *p* = 0.011). Figure 2 illustrates the comparison of AS-OCT parameters amongst the 3 groups (Table 1). CTL correlated with IT (r = 0.432, *p* = 0.031), CT (r = 0.578, *p* = 0.001), CTS (r = 0.547, *p* = 0.002), epithelial defect (r = 0.520, *p* = 0.004), infiltrate diameter (r = 0.423, *p* = 0.040) and logMAR visual acuity (r = 0.603, *p* = 0.001) at presentation, and also final logMAR visual acuity (*p* = 0.448, *p* = 0.017) (Supplementary Table 1).

### Analysis of tear cytokine and chemokine profiles

**Figure 3:**
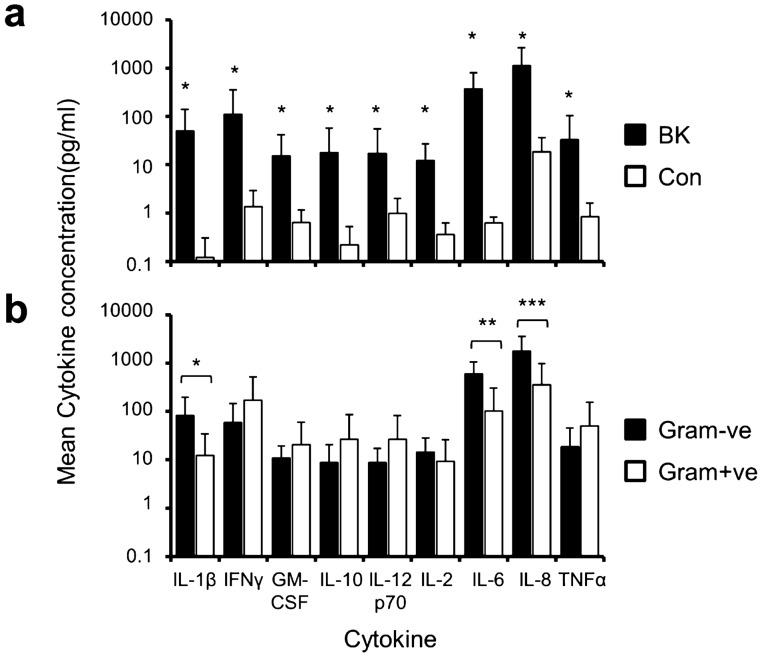
Quantification of the cytokine and chemokine levels present in tears. Comparison of cytokine and chemokines levels (pg/ml) present in tears between bacterial keratitis (BK) and control (**a**) and Gram−ve versus Gram+ve bacterial keratitis (**b**) at presentation are shown in the plots. All cytokines/chemokines were elevated at presentation of bacterial keratitis compared to controls, except for IL-12p70. The levels of IL-1β, IL-6 and IL-8 were significantly greater in Gram−ve than Gram+ve bacterial keratitis. Asterisks over the bars denote statistical significance (*p* < 0.05).

Tear samples were analysed from 13 cases of culture-positive BK, 7 cases of Gram−ve and 6 cases of Gram+ve and 5 control cases. Aetiological pathogens were *P. aeruginosa* in 6 cases, *S. aureus* in 3, coagulase-negative *Staphylococcus* in 2, coliform bacteria in 1 and *S. pneumoniae* in 1. Mean patient age was 56.7 years. Compared to controls, all cytokines/chemokines were elevated in BK except for IL-12p70 (Fig. 3a). The greatest concentration ratios of BK to controls were observed for IL-1β, IFN-γ, IL-10, IL-6 and IL-8 (Supplementary Table 2). There was a significant difference in cytokine concentrations between Gram−ve and Gram+ve BK (Fig. 3b; Table 2) for IL-1β (median [IQR]: 52.65 [84.00] vs. 2.76 [20.3] pg/ml, *p* = 0.035), IL-6 (median [IQR]: 547.91 [801.66] vs. 20.46 [163.7] pg/ml, *p* = 0.008) and IL-8 (median [IQR]: 778.88 [2682.96] vs. 120.18 [533.44] pg/ml, *p* = 0.014). There was no significant difference for IFN-γ, GM-CSF, IL-10, IL-12p70, IL-2 and TNF-α.

**Table 2:** Comparison of cytokine and chemokine levels between Gram-negative and Gram-positive human bacterial keratitis.

Cytokine	Gram−ve (pg/ml) Mean [SD] Median [IQR]	Gram+ve (pg/ml) Mean [SD] Median [IQR]	Gram−ve / Gram+ve	*p* value
IL-1β	82.67 [114.69]	12.40 [21.98]	6.7	0.035
	52.65 [84.00]	2.76 [20.3]	19.1	
IFN-γ	58.51 [86.96]	172.76 [351.90]	0.3	1.0
	22.03 [47.58]	29.72 [277.24]	0.7	
GM-CSF	10.90 [8.63]	20.64 [38.86]	0.5	0.836
	11.19 [11.92]	2.71 [34.66]	4.1	
IL-10	8.78 [11.75]	26.52 [58.57]	0.3	0.628
	4.97 [9.94]	1.47 [43.62]	3.4	
IL-12p70	8.80 [8.43]	26.80 [55.88]	0.3	0.836
	6.49 [15.74]	3.42 [41.12]	1.9	
IL-2	14.53 [13.80]	9.39 [16.63]	1.5	0.138
	10.24 [17.66]	1.92 [15.44]	5.3	
IL-6	599.62 [454.03]	102.43 [201.11]	5.9	0.008
	547.91 [801.66]	20.46 [163.7]	26.8	
IL-8	1789.48 [1785.28]	358.50 [620.71]	5.0	0.014
	778.88 [2682.96]	120.18 [533.44]	6.5	
TNF-α	18.80 [26.36]	50.12 [103.83]	0.4	0.836
	10.94 [18.26]	8.14 [82.00]	1.3	

Gram−ve Gram negative, Gram+ve Gram positive, *IQR* interquartile range.

**Figure 4:**
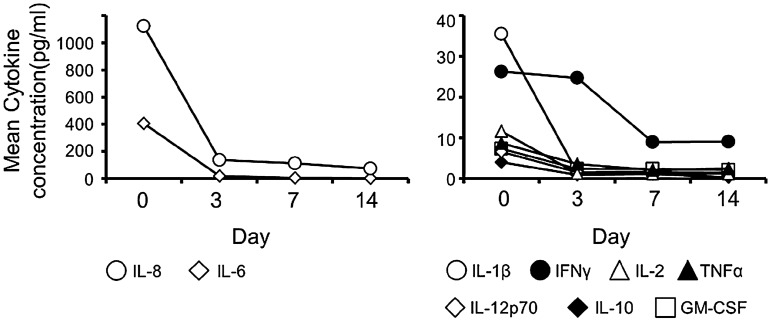
Time course cytokine and chemokine change in resolving bacterial keratitis. Quantification of IL-1β, IFN-γ, GM-CSF, IL-10, IL-12, IL-2, IL-6, IL-8 and TNF-α levels (pg/ml) at presentation and after 3, 4 and 14 days of treatment. A statistically significant reduction was found for IL-1β (*p* = 0.034), IFN-γ (*p* = 0.037), IL-2 (*p* = 0.04), IL-6 (*p* < 0.001), IL-8 (*p* = 0.004) and TNF-α (*p* = 0.008); a reduction of borderline statistical significance was found for GM-CSF (*p* = 0.068), IL-10 (*p* = 0.059) and IL12p70 (*p* = 0.068). The mean [SD] and median [IQR] values of each cytokine concentration, as well as the levels of their induction in BK compared to control tears (BK/control) at presentation and after 3, 7 and 14 days of treatment are specified in the Supplementary Table 3.

Cytokine/chemokine concentrations (n = 7) decreased significantly with treatment (Fig. 4): between presentation and day 3 of treatment there was a significant reduction for IL-1β (*p* = 0.028), IL-2 (*p* = 0.028), IL-6 (*p* = 0.018), IL-8 (*p* = 0.028) and TNF-α (*p* = 0.043) concentrations; a borderline reduction was found for IFN-γ (*p* = 0.091), GM-CSF (*p* = 0.063), IL-10 (*p* = 0.063) and IL-12p70 (*p* = 0.063) (Supplementary Table 3).

We also examined the correlation between cytokine concentrations and corneal morphology. Presentation CT showed a significantly strong correlation with IL-6 concentration (r = 0.697, *p* = 0.025) (n = 10). Strong and moderate correlations, of borderline statistical significance, were observed with IL-8 (r = 0.612, *p* = 0.06) and IL-1β (r = 0.491, *p* = 0.15) concentrations. When all examination points (n = 28) were analysed collectively, significant correlations were found for IL-6 (r = 0.635, *p* < 0.001), IL-8 (r = 0.430, *p* = 0.022) and IL-1β (r = 0.386, *p* = 0.042).

## Discussion

In this exploratory study, we recruited a clinical cohort of patients presenting with suspected bacterial keratitis. To be included for a full assessment, only patients with positive microbiology findings from corneal sampling could be analysed.

We quantified the differential acute corneal inflammatory response and tissue damage in Gram−ve, Gram+ve and microbiology−ve BK. A presentation CT ≥ 950 μm or IT ≥ 400 μm was associated with Gram−ve BK. Gram−ve bacteria induced greater corneal tissue inflammation and greater levels of IL-1β, IL-6 and IL-8 than Gram+ve bacteria. By day 3 of treatment, levels of pro-inflammatory cytokines had decreased significantly.

At presentation, CT and IT was significantly larger in Gram−ve infection. Presentation CT ≥ 950 μm was highly indicative of Gram−ve aetiology and with 100% sensitivity and 91.7% specificity, could be used as a diagnostic indicator of Gram−ve BK. However, corneal infection is a dynamic process and progression may be associated with CT reduction and IT increase^8^. Animal studies have found an increase in corneal inflammatory cell infiltration with the progression of infection^10^. A diagnostic criterion using both CT and IT may, therefore, be more applicable to BK. The criterion of CT ≥ 950 μm or IT ≥ 450 μm had 100% sensitivity and 83.3% specificity as an indicator of Gram−ve BK.

The CTs was greater in Gram−ve than Gram+ve BK by a factor of 2.3. Prior to AS-OCT, we could not accurately quantify this difference in vivo, but our findings are consistent with the large corneal infiltrates observed clinically in Gram−ve BK^6^. Bourcier et al. found that Gram−ve bacteria cause corneal infiltration of greater surface area^5^.

Our cytokine study supported the morphological AS-OCT findings, with the levels of pro-inflammatory cytokines IL-1β, IL-6 and IL-8 greater in Gram−ve than Gram+ve BK by a factor of 5 to 26.8 (Table 2). Increased cytokine levels in Gram−ve BK, probably produced from epithelial cells, fibroblasts and polymorphonuclear neutrophils (PMNs)^11–13^, may, in turn, result in greater PMN recruitment and infiltration, more corneal oedema and thus greater CTS.

The different bacterial virulence factors and activation of different pattern recognition receptors with corresponding pathways may account for the more intense inflammatory response of Gram−ve infection^14–16^. IL-1β influences PMN influx in tissues and probably produced by resident corneal cells and immune cells, such as mononuclear cells, macrophages and PMNs^20–23^. IL-1β is expressed within all corneal layers and has been detected in tears of patients with corneal infection and inflammation^24,25^.

IL-8, the cytokine with the highest absolute concentration in BK patients, probably mediates the pro-inflammatory effect of IL-1β^12^. Karthikeyan et al*.* have suggested that PMNs, as the predominant infiltrating cells in corneal ulcers, may produce most of the pro-inflammatory cytokines, including IL-8^26^. The role of IL-6 in BK is conflicted; in a study of murine *Pseudomonas* keratitis, corneal PMN recruitment was dependent on IL-6 production^27^, whereas, in a study of *S. aureus* keratitis, IL-6 knockout mice had greater levels of both bacteria and PMNs in the cornea^28^.

The host-cytokine response towards *P. aeruginosa* keratitis is mainly induced by LPS stimulation of TLR4/MD-2 and flagellin binding to TLR5. However, some strains can produce T3SS exotoxins (cytotoxic strains) that can influence the inflammatory response^29,30^. The effect of T3SS on *Pseudomonas* keratitis have been well-studied in vitro^31^*.* To our knowledge, only two major studies have examined the effect of different clinical isolates. One study using microbial isolates collected in the Steroids for Corneal Ulcers Trials (SCUT) compared the clinical characteristics of patients between invasive and cytotoxic *P. aeruginosa* strains causing bacterial keratitis. In the second study in Taiwan of isolates collected over a 10-year period. These two studies concluded that although the T3SS genotype may be a significant prognostic factor, both clinical and in vitro features were not so similarly influenced by the two genotypes^32,33^.

*S. aureus* keratitis induces the ocular inflammatory response by stimulation of TLR2^34^. Similar to *P. aeruginosa*, some *S. aureus* can secrete different virulent toxins able to damage the ocular surface. α- and γ-toxin encoding gens of *S. aureus* are present by the 95–100% of the isolates^35^. Nevertheless, it has been seen that human corneal epithelial cells respond to toxin-expressing or non-toxigenic *S. aureus* in similar ways, thus indicating that the initial epithelial cell responses are driven by toxin-independent mechanisms^36^. However, to the best of our knowledge, there is no major study using isolates collected from SCUT that compares the clinical characteristics between different S. aureus strains.

Our cytokine analysis showed that, even without corticosteroid drops, a significant reduction in the molecular markers of acute inflammation (IL-1β, IL-2, IL-6, IL-8 and TNF-α) was observed by day 3 of treatment. This finding provides a novel insight into the timing of the use of anti-inflammatory agents. Cytokines have been shown to induce the expression of matrix metalloproteinases (MMPs)^37,38^, which, together with destructive tissue enzymes and PMN can result in stromal tissue destruction^39–41^. Therefore, the use of anti-cytokine therapies in BK is likely to help limit tissue damage and prevent sight loss. Traditionally, administration of corticosteroid drops starts 5–7 days after initiation of antibiotics^2,42^. In the SCUT, administration of steroid drops began after at least 48 h of antibiotic treatment^43^. Our cytokine data suggest that the critical period for immunomodulation, to reduce tissue damage, maybe earlier than currently practised. A SCUT patient subgroup that received steroid drops within 2–3 days of starting antibiotics had approximately 1-line better visual acuity at three months compared to placebo patients^44^.

AS-OCT quantification showed that the greatest CTL was observed in Gram−ve infection. A greater tissue loss correlated with more significant corneal inflammation at presentation and also poorer final vision. This provides evidence of the detrimental effect that corneal inflammation may have on outcome in BK; infiltrating PMNs are known to release superoxide anions, hydrogen peroxide and lysosomal enzymes, which contribute to stromal destruction^16,20,45^.

We found a correlation of morphological parameters of corneal inflammation with both function and molecular markers of inflammation. These findings not only support the use of AS-OCT in clinical practice but also as a research tool investigating inflammation and tissue damage in corneal infection.

In addition to the early morphological changes, the overall time to resolution and the level of final tissue damage was more significant in Gram−ve group than the Gram+ve group, which reflects the substantial and intense inflammatory features seen initially with the Gram−ve cases.

AS-OCT provided an objective measure of microbial keratitis and assessing treatment response objectively. With the increased availability of posterior segment OCTs with anterior imaging capability, it is thus becoming an essential and routine tool in the evaluation of cornea^46^. The quantification capabilities of AS-OCT in BK have been described previously^8,9^, further work in this field, especially in the form of a multicentric prospective study, would be able to draw conclusions that could be used in everyday clinical practice. Corneal densitometry, using Scheimpflug imaging, has also been suggested as an objective measure of patient progress^47^.

Our study investigated treatment-naive patients and was conducted on early BK in order to avoid possible confounding factors in cases presenting late, such as coexisting fungal infection, prior inadequate treatment and corneal melting. A limitation of the current study was that we sampled only a small cohort of patients from the South Coast of England. Nevertheless, the most common bacteria were *Pseudomonas* and *Staphylococcus* species, in agreement with Western studies in the United States, Australia, Canada and United Kingdom^2,4,48–50^. However, wide geographic variation exists in the spectrum of bacteria involved in BK^51^. In South India, the most common bacterium is *S. pneumoniae*^52^. Thus, our findings may be applicable only to settings with a similar patient cohort. A future multi-centre study to include a broader range of pathogenic bacterial species would help to confirm our findings. The approach could also be used in studies using bacteria with detailed strain classification. This could help to detect whether the clinical phenotype is dependent on strain variations.

In our Gram+ve cohort like other studies, a high number of coagulase-negative staphylococcal species (CNS)^53–55^, majority of which are *S. epidermidis*^4^. CNS represent over 80 different species and are regular isolates worldwide^53,54^ and are increasingly found to be responsible for infections and disease pathogenesis^53^. Similarly, our regional laboratory has shown that the majority of the CNS isolates are *S. epidermidis*^4^. To meet the inclusion criteria for our study, patients needed to present with clinical infection with a corneal infiltrate measuring > 2 mm in diameter (see methods). Corneal sampling was performed using an aseptic approach by a corneal ophthalmologist. Additionally, these cases resolved with antibiotic therapy. Therefore, the positive isolation of CNS organisms and the subsequent response to antibiotic treatment indicates an infectious aetiology'.

In summary, this exploratory study shows a range of non-invasive clinical parameters using OCT and tear cytokine measurements in response to BK. This approach can distinguish BK cases caused by Gram−ve and Gram+ve organisms in a clinical cohort. Greater inflammation and corneal tissue loss were observed in BK cases with Gram−ve bacteria. AS-OCT measurements of CT and IT were useful diagnostic indicators, and a CT ≥ 950 μm or IT ≥ 450 μm were highly indicative of Gram−ve BK. Pro-inflammatory cytokine levels also varied between Gram−ve and Gram+ve pathogens with a large reduction occurring by day 3 of treatment. These findings form a basis for a larger-scale clinical evaluation of this approach to BK. Additionally, the study also indicates that there is an earlier time window prior to day 3 for immunomodulatory intervention.

## Methods

### Recruitment of patients, inclusion criteria and healthy controls

Patients presenting over a 2-year period to Southampton Eye Unit were prospectively recruited. The inclusion criterion was untreated BK, diagnosed clinically by a typical history and epithelial ulceration with stromal inflammatory infiltration^8,9,47^ of greater of 2 mm on examination. Exclusion criteria were ulceration at risk of perforation, symptoms greater than 14 days, neurotrophic or blind eye, the revision to non-BK or previous corneal infection. Resolution of infection was accepted when the epithelial defect and inflammation was resolved. Healthy controls, with no prior ocular history, and normal slit-lamp examination were recruited. Protocols were approved by the National Research Ethics Service (NRES)—Berkshire 06/Q1602/56 and patients informed consent was obtained in accordance with the Declaration of Helsinki.

### Clinical and slit-lamp examination

Clinical assessment and slit-lamp examination were done at presentation and during treatment. Data on patient demographics, infection risk factor, co-morbidity, symptoms duration, logMAR visual acuity (VA), slit-lamp dimension (average of longest diameter and its perpendicular) of the epithelial defect (ED), slit-lamp corneal infiltration (average of horizontal and vertical) diameter (ID) and aetiological pathogen were collected.

### Microbiology procedures

Samples were taken by experienced corneal ophthalmologists. Corneal scrapes were performed under aseptic conditions and microbiology diagnosis based on standard Public Health England microbiology services^56^. These services provide microscopy within 24 h and culture of the different specimens in differential agar medium in time of 48 to 72 h^56^.

### Anterior segment optical coherence tomography

Visante® OCT (Carl Zeiss Meditec Inc, CA, USA) corneal imaging was done at presentation and after the resolution of infection; a standardised scanning protocol with IT and CT measurements were used (Fig. 1)^9^. Control subjects had bilateral four-quadrant high-resolution AS-OCT scans at 0°, 45°, 90° and 135°, dividing each cornea into eight pie segments. Each segment was divided into a central 4 mm area, a paracentral area defined by the 4 mm zone and an outer 8 mm zone, and a peripheral area extending from the 8 mm zone to the limbus. CT was measured in the centre of each area with calliper tools for all controls, and the mean CT of each area mapped (Fig. 1).

A parameter of corneal inflammation, termed corneal tissue swelling (CTS), was defined as the difference between the patient's CT at presentation and the mean control CT, obtained from the area of the plotted CT maps corresponding to the location and eye laterality of the patient's corneal ulcer. Corneal tissue loss (CTL) was defined as the difference between the patient's final CT after the resolution of infection and the mean control CT, also obtained from the area of the CT maps corresponding to the ulcer location and laterality.

Patients were treated with topical antibiotics. Ofloxacin (0.3% w/v) and cefuroxime (5% w/v) drops were used hourly for 48 h and then reduced to hourly during waking hours for the next 24 h (6 am to 12 am). After 72 h, antibiotics were reduced to 2-hourly for the next five days. After day 3 (72 h) Steroid drops were used after day 3 (72 h), once bacterial growth was confirmed, the dosage of prednisolone 0.5% 2-hourly for five days. Cases were categorised into Gram−ve, Gram+ve and microbiology negative (no bacteria identified on microscopy or culture).

### Tear cytokine analysis

Patients during the second half of the study had tear sample collection at presentation, and on days 3 (before steroid treatment), 7 and 14 of treatment; healthy controls had tears collected on one occasion. Samples were collected by conjunctival lavage of the affected eye without topical anaesthesia. Sterile normal saline (50 μL) was infused at room temperature into the lower conjunctival sac by gently pulling down the lower eyelid and with a needle-free insulin syringe. After 2 s, the lavage fluid was aspirated back into the same syringe without the syringe touching the conjunctiva. The lavage was repeated, and the two syringes were sealed and transferred on ice to the laboratory. The conjunctival fluid from the two syringes was pooled and spun down in a centrifuge (400×*g* for 10 min at 4 °C), and the supernatants were analysed using the pro-inflammatory multiplex assay kit from Meso Scale Discovery (Gaithersburg, MD, USA) for levels of the following cytokines and chemokines: IL-2, IL-8, IL-12p70, IL-1β, GM8, CSF, IFN-γ, IL-6, IL-10, and TNF-α^57^.

### Statistical analysis

As this study, is classed as a hypothesis-generating study and has been designated "exploratory" according to the National Institute for Health Research (NIHR) regulations (https://www.nihr.ac.uk/documents/nihr-research-for-patient-benefit-rfpb-programme-guidance-on-applying-for-feasibility-studies/20474). Statistical methods were done following the Statistical Analyses and Methods in the Published Literature (SAMPL) guidelines^58^.

LogMAR VA, ED, ID, IT, CT, CTI and CTL values were compared between Gram−ve, Gram+ve and microbiology negative groups. Cytokine concentrations were compared between Gram−ve and Gram+ve groups only. The correlation between parameters was investigated. The sensitivity, specificity, positive predictive value (PPV) and negative predictive value (NPV) of increased CT as a diagnostic indicator of Gram−ve infection were examined. We have previously found a mean difference between *Pseudomonas* and non-*Pseudomonas* BK of 265 μm (mean [SD] CT: 1072 [196] vs. 807 [142] μm)^9^. For OCT studies, a sample size of 11 patients in each group would provide 90% power to detect a smaller difference of 200 μm between a Gram−ve group and Gram+ve group at a significance level of 0.05.

Data normality was assessed by Shapiro–Wilk statistics and histograms. IT, CT and CTS showed a normal distribution and were analysed with ANOVA, Dunnett and paired t-tests; all remaining variables, including cytokine concentrations, had a skewed distribution. Non-normal data were analysed with Kruskal Wallis, Mann–Whitney U, Wilcoxon signed-rank tests and Friedman's two-way analysis of variance. The Spearman rank test was used for correlation analysis between cytokine concentrations and morphology on the group that had both OCT and tear cytokine analysis. Where multiple simultaneous statistical tests were used, appropriate Bonferroni corrections were made.

The Statistical Package for Social Science (IBM SPSS Statistics for Macintosh, Version 22.0. Armonk, NY: IBM Corp) was used; *p* < 0.05 was considered significant and marked with an asterisk (*).

## Supplementary information

Supplementary Tables.
