# Author Correction: Morphological and cytokine profiles as key parameters to distinguish between Gram-negative and Gram-positive bacterial keratitis

**DOI:** 10.1038/s41598-021-85007-w

**Published:** 2021-02-26

**Authors:** Aris Konstantopoulos, Maria del Mar Cendra, Michael Tsatsos, Mariam Elabiary, Myron Christodoulides, Parwez Hossain

**Affiliations:** 1 Southampton Eye Unit, MP104, Southampton General Hospital, University Hospital Southampton NHS Foundation Trust, Tremona Road, Southampton, SO16 6YD UK; 2 Clinical and Experimental Sciences, Southampton General Hospital, University of Southampton, Southampton, UK; 3 Aristotle University of Thessaloniki, Thessaloniki, Greece; 4 Eye Department, Dorset County Hospital NHS Foundation Trust, Dorchester, UK

Correction to: *Scientific Reports* 10.1038/s41598-020-77088-w, published online 18 November 2020

The Acknowledgements section in this Article contains an incomplete funding name.

“The Royal College of Surgeons of Edinburgh”.

should read:

“The Royal College of Surgeons of Edinburgh and The Royal Blind”.

