# Supplementary material for: Morphological and cytokine profiles as key parameters to distinguish between Gram-negative and Gram-positive bacterial keratitis
Source: Sci Rep. 2020 Nov 18;10:20092. doi: 10.1038/s41598-020-77088-w (PMC7674500; doi:10.1038/s41598-020-77088-w)
Supplement: Supplementary file 1 — Supplementary Tables. [file 41598_2020_77088_MOESM1_ESM.pdf]

Morphological and cytokine profiles as key parameters to distinguish between Gram-negative and Gram-positive bacterial keratitis

Aris Konstantopoulos[1,2]*, Maria del Mar Cendra[2]*, Michael Tsatsos[3], Mariam Elabiary[1], Myron Christodoulides[2], Parwez Hossain[1,2#]

Supplementary Information

Supplementary Table 1. Correlation analysis of Corneal Tissue Inflammation and Corneal Tissue Loss

	CTI		CTL	
**Parameter**	**Spearman r**	***p*-value**	**Spearman r**	***p*-value**
Presentation logMAR VA	0.610	<0.001	0.603	0.001
Presentation epithelial defect	0.553	<0.001	0.520	0.004
Presentation infiltrate diameter	0.693	<0.001	0.423	0.040
Presentation infiltrate thickness	0.662	<0.001	0.432	0.031
Presentation corneal thickness	NA		0.578	0.001
CTI	NA		0.547	0.002
Final corneal thickness	0.610	<0.001	NA	
Final logMAR VA	0.090	0.577	0.448	0.017
CTL	0.547	0.002	NA	

CTI: corneal tissue inflammation, CTL: corneal tissue loss, r: correlation coefficient, VA: visual acuity

Supplementary Table 2: Cytokines and chemokines concentration in the tears of BK patients compared to controls

Cytokine	BK (pg/ml) Mean [SD] Median [IQR]	Controls (pg/ml) Mean [SD] Median [IQR]	BK/Controls	*p*-value
IL-1β	50.24 [90.04]<br>9.85 [60.64]	0.12 [0.19]<br>0.00 [0.30]	419<br>NP	<0.001
IFN-γ	111.24 [242.68]<br>25.40 [12.78]	1.35 [1.57]<br>0.71[3.00]	82<br>36	0.001
GM-CSF	15.39 [26.31]<br>8.82 [12.78]	0.64 [0.52]<br>0.57 [0.82]	24<br>15	<0.001
IL-10	17.97 [39.79]<br>4.70 [8.76]	0.22 [0.30]<br>0.02 [0.54]	82<br>235	0.003
IL-12p70	17.10 [37.73]<br>5.77 [12.06]	0.98 [1.05]<br>0.77 [1.54]	17<br>7	0.10
IL-2	12.16 [14.75]<br>7.62 [15.24]	0.36 [0.27]<br>0.41 [0.82]	34<br>19	<0.001
IL-6	370.15 [431.83]<br>205.18 [609.04]	0.63 [0.20]<br>0.58 [1.16]	587<br>354	<0.001
IL-8	1129.03 [1518.37]<br>358.77 [2197.64]	18.54 [17.98]<br>15.02 [32.74]	61<br>24	0.001
TNF-α	33.25 [71.44]<br>9.98 [18.98]	0.84 [0.78]<br>0.86 [1.72]	40<br>12	0.007

BK: bacterial keratitis, IQR: interquartile range, NP: not possible

Supplementary Table 3. Concentrations cytokines and chemokines from presentation to days 3, 7 and 14 of treatment

Cytokine	Day 0	Day 3	Day 7	Day 14	
Cytokine concentration (pg/ml)					
	Mean [SD]<br>Median [IQR]<br>BK/controls	Mean [SD]<br>Median [IQR]<br>BK/controls	Mean [SD]<br>Median [IQR]<br>BK/controls	Mean [SD]<br>Median [IQR]<br>BK/controls	*p*-value
IL-1β	35.47 [36.08]<br>15.83 [62.74]<br>295.6	1.60 [1.03]<br>1.89 [2.08]<br>13.3	1.69 [1.35]<br>1.69 [2.68]<br>14.1	1.35 [1.44]<br>0.55 [2.62]<br>11.3	0.034
IFN-γ	26.26 [18.51]<br>22.03 [30.94]<br>19.5	24.74 [13.42]<br>12.04 [23.84]<br>18.3	8.99 [9.89]<br>7.62 [12.08]<br>6.7	9.08 [7.09]<br>7.92 [10.36]<br>6.7	0.037
GM-CSF	7.37 [5.23]<br>8.82 [9.76]<br>11.5	2.35 [1.57]<br>1.97 [2.94]<br>3.7	2.39 [1.88]<br>1.70 [3.84]<br>3.7	2.08 [1.39]<br>1.74 [1.94]<br>3.3	0.068
IL-10	4.06 [3.16]<br>4.70 [4.94]<br>18.4	0.92 [1.21]<br>0.42 [2.64]<br>4.2	1.11 [1.56]<br>0.31 [1.96]<br>5.0	0.38 [0.43]<br>0.24 [0.56]<br>1.7	0.059
IL-12p70	6.62 [7.78]<br>5.77 [4.96]<br>6.8	1.60 [1.12]<br>1.14 [2.02]<br>1.6	1.29 [1.28]<br>0.85 [2.16]<br>1.3	1.44 [1.27]<br>1.13 [1.00]<br>1.5	0.068
IL-2	11.60 [13.98]<br>7.62 [9.14]<br>32.2	1.32 [0.98]<br>0.99 [1.90]<br>3.7	1.18 [1.06]<br>0.89 [0.70]<br>3.3	1.22 [0.84]<br>0.87 [0.88]<br>3.4	0.04
IL-6	407.94 [359.38]<br>244.94 [576.46]<br>647.5	18.81 [26.51]<br>8.55 [26.00]<br>29.9	5.31 [5.14]<br>3.05 [7.48]<br>8.4	2.68 [2.90]<br>1.62 [2.40]<br>4.3	<0.001
IL-8	1122.52 [1321.48]<br>363.46 [2770.90]<br>60.5	137.13 [77.84]<br>124.10 [84.76]<br>7.4	113.07 [141.77]<br>55.99 [175.50]<br>6.1	73.91 [77.84]<br>30.43 [138]<br>4.0	0.004
TNF-α	8.75 [6.71]<br>6.31 [7.48]<br>10.4	3.59 [3.04]<br>2.79 [6.44]<br>4.3	2.08 [2.12]<br>1.71 [2.30]<br>2.5	2.44 [1.70]<br>1.96 [2.20]<br>2.9	0.006

BK: bacterial keratitis, IQR: interquartile range
